# User needs and design features of sanitation digital solutions in Kawempe division, Kampala Uganda: a user centered design approach

**DOI:** 10.3389/fpubh.2023.1107604

**Published:** 2023-12-06

**Authors:** Comfort Hajra Mukasa, Maureen Nankanja, Mtwalib Walude, Maurice Muzini, Patrick Kagurusi

**Affiliations:** Amref Health Africa, Kampala, Uganda

**Keywords:** sanitation, digital, solutions, SaniDigS, Uganda

## Abstract

Despite improvements in access to water and sanitation services globally, a significant population in Sub Saharan Africa has limited access to improved sanitation facilities. Furthermore, there is lack of a centralized digital platform for data exchange among stakeholders for sanitation services planning and provision in Uganda. A user centered design (UCD) approach was used to develop and pilot a one stop sanitation digital solutions (SaniDigS) center in Kawempe division, Kampala, Uganda. This involved three phases (1) understanding the sanitation status of the community which involved interviewing 1,844 household heads, (2) specifying the user needs of the proposed digital solution through stakeholder meetings and (3) Co designing of the innovations with potential users through stakeholder workshops. The quantitative data was visualized through the SaniDigS platform dashboard. The stakeholder meeting transcripts and notes were thematically analyzed to identify the user needs. The community user needs included an innovation that ensures confidentiality, is low cost and user friendly without internet connection. The service provider needed a sanitation digital solution that can market their sanitation products while, policy makers and planners needed comprehensive, real time data collection and sharing for trend analysis and informed decision making. The codesigned features of the SaniDigS informed by the user needs included: The SaniDigS Champion Application, SaniDigS platform dashboard, SaniDigS mobile application and the call center. The community in Kawempe showed need for subsidized sanitation products and we recommend further research to evaluate the effects of SaniDigS on planning, coordination, and access to sanitation services.

## 1 Introduction

The use of improved sanitation facilities has increased over the years from 28% in 2000 to 45% in 2017, and the availability of hand-washing facilities has also increased to 60% worldwide (1, 2). Despite this progress, an estimated 4.5 billion people still have no access to improved sanitation facilities, of which 2.3 billion have no access to basic sanitation facilities and 892 million practice open defecation (1). Additionally, only 25% of the population in Sub-Saharan Africa have access to hand-washing facilities with one in four people being able to access hand washing facilities with water and soap (1). In Uganda, the percentage population accessing basic sanitation services in rural areas is 18% and 44.8% in urban areas in the year 2019 (3). This leaves the country with a big gap of ensuring that everyone has access to a basic sanitation facility by 2030 in line with the Sustainable Development Goal (SDG) 6.2 which Uganda is committed to (3). In Kampala City-the capital of Uganda, it was revealed that 38% of latrines constructed have unlined pits that leak into underground aquifers, which has contributed to cholera outbreaks in the city (4).

The low access to basic sanitation coverage in Uganda has been attributed to a number of factors including technological challenges ranging from lack of knowledge of context specific appropriate technology, to lack of awareness of the available sanitation services within reach of the consumers. Additionally, there is uncoordinated planning and service provision which is manifested by 30% of the emptied fecal sludge in city being emptied directly into the low lying environment and inappropriate latrine designs being promoted by different stakeholders (4, 5).

Furthermore, there is also lack of a centralized digital platform for data exchange among stakeholders for sanitation services provision in Uganda, with data being collected manually (Paper based) which makes it tedious to verify and triangulate information (6). The lack of real-time digital data undermines sanitation service planning and provision, and the value chain system's ability to identify and quantify timely sanitation needs for effective planning and service delivery thus impinging on efforts to reach everyone at all time. Additionally, due to lack of real-time digital data, sanitation service providers are disconnected from the community, with the majority of the community members not knowing where to access sanitation services such as latrine emptying and products such as bio digesters. There is need for an improved mechanism to coordinate sanitation data collection and exchange to facilitate more efficient and effective sanitation policy-and-decision-making.

Therefore, Amref Health Africa Uganda aimed to design and implement a one stop sanitation digital solutions (SaniDigS) center and test if timely access to sanitation information leads to improved sanitation planning, increased access to safely managed sanitation services and improved multi-sectoral collaboration (7). The SaniDigS center provides: an application for digital data collection, a comprehensive dashboard for visualization of data, a mobile app which connects services providers and products to the clients and a call center to answer client requests and link them to products and service providers. SaniDigS was implemented in Kawempe division a division that is characterized by a high water table and frequent flash floods that impact sanitation (8). In this division, 78% of the community members are tenants that cannot make a decision on the form of sanitation facility that they should have (9).

The innovation involved creating a single digital platform for all sanitation service providers for constructive engagement with consumers, digitalization of all demand and service provision processes for real-time service provision. The theory of change is that the innovation facilitates equitable allocation of resources, better planning, evidence-based decision making; formalizes practice and standardizes service provision; thereby, improving effective and efficient sanitation service provision.

This paper adopted a user centered design approach to document SaniDigS implementation, user needs and design features of the SaniDigS. This will contribute to knowledge on the best practices in designing of sanitation digital solutions and hopefully stimulate further research on digital solutions for sanitation.

### 1.1 Context—setting and population

The SaniDigS innovation was implemented in Kawempe division, Kampala city, Uganda. Kawempe division is located in the Northern part of Kampala city and it has the largest number of informal settlements in the city. The division has an estimated population of 338,665 people, 21 parishes and 112 zones (10). The Division medical department is the duty bearer for sanitation service provision across the 112 zones. There are 4 other non-government organizations (NGOs) in this division that offer sanitation services to the people. The private sector has also been attracted to this division to mostly offer sanitation products and latrine emptying services (4). All these service providers are supposed to be coordinated by the division health office (4). According to the Kampala Capital City Authority (KCCA) statistical abstract of 2019, 53% of the people in Kawempe division are youth. In addition, 73% of the population are women and children below 15 years of age (11). According to the Kampala City wide sanitation mapping of 2017, 81% of the population of Kawempe are residents within Kyebando parish. Of all the residents in Kawempe, 78% are tenants who live in low lying areas characterized by congestion and poor drainage systems. The main form of sanitation access in the division is pit latrines at 94% while the rest have septic tanks and/or sewer connection with 2% of the population still practicing open defecation (12). The division is also home to Mulago National referral hospital and the oldest University in Uganda- Makerere University. The main economic activity in the area is trade, both small and medium size businesses. The primary study population was community consumers of sanitations services. The study also included technical persons and local artisans involved in delivering sanitation services as well as urban authorities who participate in coordination and planning of sanitation services.

### 1.2 Author roles

The authors played diverse roles in the project, with CHM serving as the principal investigator, MN as the project data analyst, MW as the co-principal investigator, MM as the technical lead, and PK as the project coordinator.

## 2 Methods

**Figure 1:**
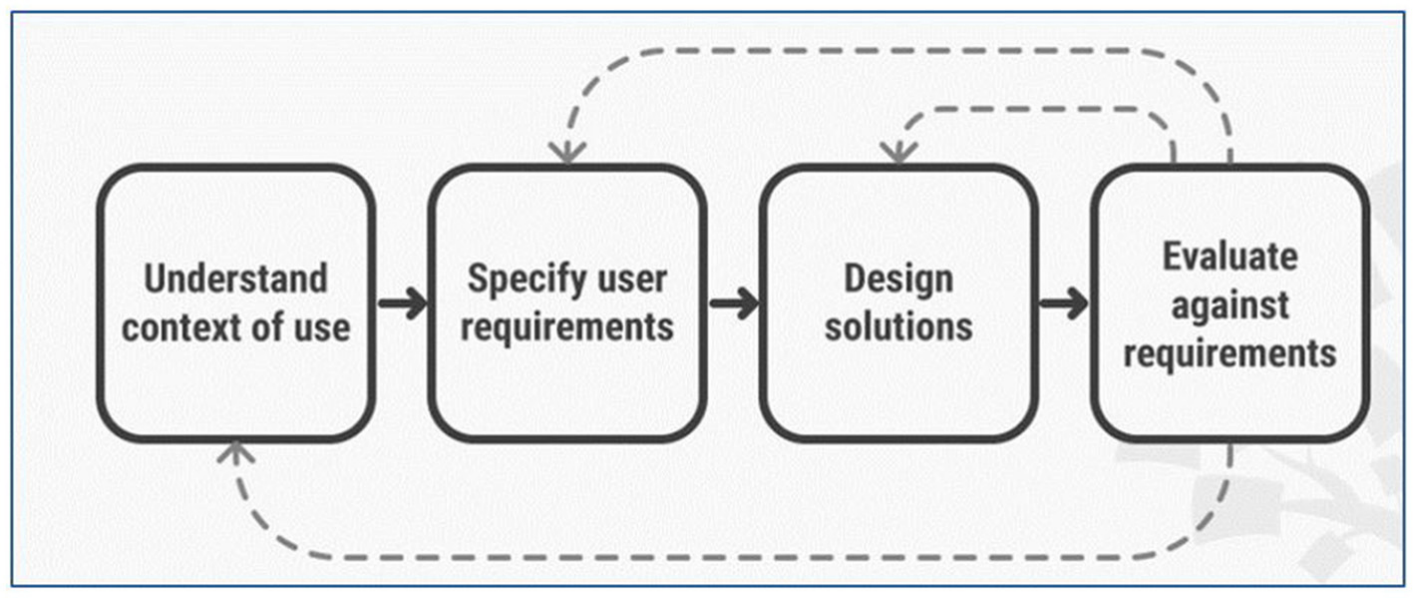
User centered design phases (13).

A user centered design (UCD) approach was used to develop and implement SaniDigS. User-centered design is an iterative process that focuses on an understanding of the users and their context in all stages of design and development. This involves four phases (1) understand the *context* in which users may use a system, (2) identifying and specifying the users' *requirements*, (3) a *design* phase in which the design team develops solutions and (4) an *evaluation* phase which involves assessment of outcomes of the evaluation against the users' context and requirements, to check how well a design is performing (13, 14) (see Figure 1).

In this paper, we present the data collection process across the first three phases of the UCD approach as were applied in SaniDigS. This approach has been recommended by earlier scholars (15).

### 2.1 Phase 1: context

To understanding the context, a household survey was conducted in Kawempe division in order to assess the baseline sanitation status of the community. The sample size for household survey was determined using the Taro Yamane's formula (16). A total of 1,844 households were included in the survey. Stratified random sampling procedure was followed to select respondent. First, all 19 parishes in Kawempe division were listed. Using a computer algorithm, 2/19 parishes were randomly selected. From the 2 parishes, a total of 80 villages were listed and using a computer algorithm, 10/80 villages were randomly selected. At village level, systematic sampling was applied by the research team moving through imaginary concentric circles to sample every 5th household. For the selected household, a household head was interviewed.

Between 17th November and 17th December 2020, the household survey data was collected by a team of 2 research assistants, 5 health assistants and 20 local artisans/sanitation promoters who were trained for 4 days. The data collection team conducted a pre-test of the household questionnaire for 5 days. Thereafter, data was collected using an electronic structured questionnaire. The data collection tools were uploaded onto smart phones using the Open Data Kit (ODK) to facilitate real-time uploading of the collected data onto the dashboard, real-time response to data quality issues and real-time analysis of trends.

The household survey outcome variable was sanitation status. This was measured using several indicators including: latrine coverage in the sample, hand washing coverage in the sample, common sanitation and hygiene practices reported by the respondents. Independent variables included socio demographic characteristics of household heads (sex, employment, literacy, household occupancy, HIV status and common illnesses that affected the household members). We also collected information on the perceived importance of sanitation digital solutions, user needs and design features of a hypothetical sanitation digital solution. The data was summarized in frequency tables.

### 2.2 Phase 2: user needs

To identifying user needs as recommended by (15, 17), we completed analysis of data from the household survey described above and the findings fed into the next phase of stakeholder engagement. We conducted one stakeholder workshop with 30 key stakeholders including the community members, village health teams (a cadre of village level health workers), policy makers including representatives from the ministry of health, ministry of water and environment, Kampala capital city planning officers from department of health and services providers including small scale sanitation business men and women. These were purposively selected based on their knowledge, experience and work in sanitation planning and provision.

The workshop was conducted on 28th April 2021 and the workshop proceedings kicked off with a presentation of preliminary findings from the household survey followed by a facilitated discussion. Using a stakeholder meeting tool with key guiding question, participants were asked to reflect on possible sanitation digital solutions that could meet user needs in solving sanitation challenges in Kawempe. The participants were also asked to validate the design features that would make up a good sanitation digital solution. The discussion was tape recorded with consent from the participants. Analysis was done by manifest content analysis to generate a list of user needs for a sanitation digital solution. Quotes were presented to illustrate emerging themes from the stakeholder workshop.

### 2.3 Phase 3: design

To co-design the innovation, the research team from Amref Health Africa (Amref) held another stakeholder meeting with potential “solution users” (community members, village health teams and service providers including small scale sanitation business men and women) engaged in phase 2 to co-design and develop SaniDigS. The user needs and design features proposed in phase 2 were imbedded in the co-design phase Two prototyping workshops were conducted to co-design the innovation and were attended by 5 participants. This involved one (1) village leader (from Kyebando Central village), to observe and interpret the WASH indicators concerning the village; one (1) Village Health Team (VHT)/Community Health Worker (CHW) to rally and recruit households to participate in project activities including: purchasing of WASH products/services and sharing household WASH data. Additionally, we had a WASH product provider (supplying briquettes) to forecast and observe the opportunity in supplying renewable cooking fuel in form of briquettes to participating households and a Healthcare superintendent (from Kampala City Council Authority, KCCA) to provide a quality check on the WASH data collected against historical relevant data in the village and share reports. Lastly, we had a Non-Governmental Organization (NGO) called Milele Foundation to support in community participation of Women and Girls in the project to exploit low hanging fruit WASH opportunities including making bags from waste (tarpaulin) for school children and more.

From the co-design phase a work flow chart was generated to demonstrate the elements of a user informed sanitation digital solution and the information flow in the solution. The workflow involved four steps; (1) understanding users and their needs, (2) defining user goals and tasks, (3) prototyping and testing the design with the users, (4) Evaluation to ensure the design meets the needs of all stakeholders as guided by (18). In detail, during the development of the SaniDigS solution, key beneficiaries of the solution were engaged at the different levels of iteration of the System Development Life Cycle (SDLC). Before prototyping of the solution, steering committee meetings were held regularly online with policy makers, sanitation businesses, and Kyebando parish leadership who made their expectations clear on their demands and those of the community. Upon SaniDigS prototype development to the level of satisfaction of the committee, the parish inhabitants were surveyed at household level with approved digital sanitation tools to test the assertions made. It is at this stage that impact of factors mainly of price, time, customer satisfaction, technology access, and income level were captured to support the next iteration of the SaniDigS solution as recommended by existing literature (19, 20). At the end of each iteration stage, we trained the Village Health Teams (VHTs) to support in the deployment by encouraging users/potential clients during the onboarding process to download and install the application and access the feedback module to submit their expectations. This solution workflow was used to refine SaniDigS, set up an application and dashboard.

## 3 Findings

The results are presented across the three phases of the UCD approach.

### 3.1 Phase 1: context

#### 3.1.1 Background characteristics of household survey respondents

Majority 1,386/1,844 (75.2%) of the respondents were females, slightly less than half 728/1,844(47.3%) were married. The main form of business operation was running a shop 1,083/1,844(58.7%), 694/1,844(41.3%) had attained secondary education level, and the majority 1,539/1,844(83.5%) of the households did not have dependents. At household level, 148/1844(8.0%) households had Persons living with Disabilities (PWDs) and 55/1,844(3.0%) were connected on a public sewer network (Table 1).

**Table 1:** Background characteristics of the respondents.

**Variable**	**Frequency (*N* = 1,844)**	**Percentage (%)**
**Sex**		
Male	458	24.8
Female	1,386	75.2
**Marital status (*****n*** = **1,538)**		
Married	728	47.3
Single	467	30.4
Engaged	137	8.9
Widowed	101	6.6
Divorced	61	4.0
Minor	44	2.9
**Households with PWDs (*****n*** = **1,844)**		
Yes	148	8.0
No	1,696	92.0
**Households on Sewer networks (*****n*** = **1,844)**		
Yes	55	3.0
No	1,789	97.0
**Forms of Business owned**		
Shops	1,083	58.7
Restaurants	35	1.9
Hair saloon	279	15.1
Bodaboda	8	0.4
Bar	57	3.1
Others	382	20.7
**Literacy levels**		
None	342	20.4
Primary	477	28.4
Secondary	694	41.3
Tertiary	167	9.9
**HIV status n** = **1, 007**		
Negative	945	93.8
Positive	62	6.2
**HH has dependents**		
Yes	305	16.5
No	1,539	83.5

#### 3.1.2 Community sanitation status

The majority 1,029/1,844 (57.1%) of the households were using an ordinary pit latrine, 85.8% of the households did not have a hand washing facility, less than half 24.5% of the sanitary facilities were full and the majority (78.3%) of the households were sharing the sanitary facilities (Table 2).

**Table 2:** Community sanitation status.

**Variable**	**Frequency (*N* = 1,844)**	**Percentage (%)**
**Type of sanitary facility used**		
Ordinary pit latrine	1,029	57.1
VIP	526	29.2
Pour flush to septic tank	65	3.6
Pour flash to sewer	57	3.2
Open defecation	38	2.1
Compost latrine	32	1.8
Toilet without superstructure	27	1.5
Toilet with temporary superstructure	19	1.1
**Hand washing facility**		
Yes	240	14.2
No	1,449	85.8
**Status of sanitary facilities**		
Full	414	24.5
No	1,276	75.5
**Sharing sanitary facility**		
Yes	1,379	78.3
No	378	21.5
NA	5	0.3
**Number of HH latrine stances**		
1	1,039	56.3
2 and more	805	43.7

### 3.2 Phase 2: user needs

#### 3.2.1 User needs of a sanitation digital solution

Several user needs were highlighted by the stakeholders during the consultation that fed into phase 2 of the UCD approach (identifying user needs). These user needs (the characteristics the solution should have in terms of scope, service, and product) are summarized in Table 3 per stakeholder category.

**Table 3:** Summary of SaniDigS user needs.

**Key stakeholders**	**User needs**
Users/Community member	• Low cost of services/products • Ease of use of the digital platform • Good quality products/services • Confidentiality and privacy • Timely service provision • Accessible offline and without an email address
Policy makers and implementers	• Easy to use • Comprehensive data collection • Ability to analyze and visualize • Linked to the national health management information system (HMIS) • Scalable • Able to track sewerage connections
Service Providers	• Market the sanitation products • Connected to customers • An advertisement platform for sanitation business products

The community members noted that they wanted a sanitation digital solution that could enable them to report their sanitation situation with anonymity, a system that would enable them to get cheap services in the shortest time possible, a system that could enable them follow up in case there is delay in service provision and a system that was user friendly to a lay person. These user needs were expressed in the quotes below:

“*…. the platform should have products and services that are at a lower price that attracts clients to purchase and utilize the services...”* (**Community member 1**)

“*An ordinary person should be able to benefit even if they do not have a personal email account, or even internet…”* (**Community member 2)**

“*The quality of sanitation products that we purchase through the platform need to be of good standard”* (**Community member 3**)

The service providers who include the small-scale sanitation business owners highlighted that the sanitation platform should be able to help market their (business) sanitation products, provide linkage to customers and use the platform for advertising sanitation products.

One of the service providers noted:

“*...We (service provider) should be able to get orders for our services on the platform…. The platform should be able to attract a market for our sanitation products”* (**Service provider 1**).

Another service provider highlighted that:

“*…the platform should have the ability to keep track of sales and the business referrals made to enable payment of commissions”* (**Service provider2**).

The policy makers and planners expressed the need for a digital solution platform that can enable comprehensive, real time data collection and sharing for trend analysis in order to inform decision making. They also noted that the innovation should be easy to use in conducting automatic analytics and data visualization. Some of the policy makers noted that the digital solution should be linked to the HIMS and collect more than just sanitation data, be easily scalable to other small towns and be able to track sewerage connections and management.

### 3.3 Phase 3: the co-design

#### 3.3.1 The co-design of sanidigs and its design features

From phase 3 of the UCD approach, through the stakeholder consultations and prototype workshops, we co-designed a one stop sanitation digital solutions (SaniDigS) platform. As indicated by the findings, the platform aimed to (1) eliminate the middlemen to reduce costs on sanitation products, (2) provide a solution for the lower level government officials to be able to anonymously raise WASH-related concerns, queries or alerts that could possibly cause them issues with their bosses, (3) the service providers wanted to have a business but without the need to pay rent, therefore, having the option of doing business online on one platform fulfilled that need, (4) the government officials wanted to have real time data and (5) the NGO involved wanted to see what other opportunities would come out of this in form of requests from the community.

The solution, therefore, was designed as a system and platform with a customer application, the service provider application and a data platform (dashboard) to be able to cater for the various user's needs as identified in phase 2. The Platform is a real-time data collection and management platform that automates processes focused on sanitation information and service provision. The SaniDigS platform is hosted on a cloud platform to support fast data manipulation, analytics, machine learning and artificial intelligence. The cloud platform allows enrichment of data with patterns and insights to facilitate opportunity exploitation, problem identification and solving. SaniDigS has the following features: (1) The SaniDigS Champion Application for data collections, (2) The SaniDigS platform dashboard for data visualization (3), The SaniDigS mobile application which connects the service providers and the community and (4) The call center which handles any inquiries and also links the community to service providers. The SaniDigS project followed the guidelines of development from the Digital Public Good Alliance (DPGA) (21). SaniDigS is listed on DPGA registry (2). SaniDigS is developed under the GNU General Public License version 3 (22). The project adheres to privacy and other applicable international and domestic laws including: General Data Protection Regulation (GDPR) in the European Union, the WHO guidelines (19) and the republic of Uganda, the data protection and privacy act, 2019 (23). The project has taken the following steps to ensure adherence to the laws including; Institution Review Board (IRB) project review, clearance from the Uganda National Council for Science & Technology (UNCST) for research and data collection; Privacy policy- https://docs.google.com/document/d/1WE3aUn0uLhVWz-z6-R0PKbJ1Kr66IgNc/edit?usp=sharing&ouid=116864198190504455367&rtpof=true&sd=true.

Terms & conditions https://docs.google.com/document/d/1aRX5h5056YE7f6CIrHujf6Lt_RAMn75a/edit?usp=sharing&ouid=116864198190504455367&rtpof=true&sd=true.

Terms of use https://docs.google.com/document/d/1WUjQSoo_A1G5h2kEtCX7Vk1TigPrWEna/edit?usp=sharing&ouid=116864198190504455367&rtpof=true&sd=true.

The SaniDigS project offers its development tools and technologies available on Github: https://github.com/Amref-Health-Africa-in-Uganda/SaniDigSMobile.

After the User Center Design (UCD), the SaniDigS solution was piloted in Kyebando parish. Below we describe each of the design features and the associated work and information flow.

**Figure 2:**
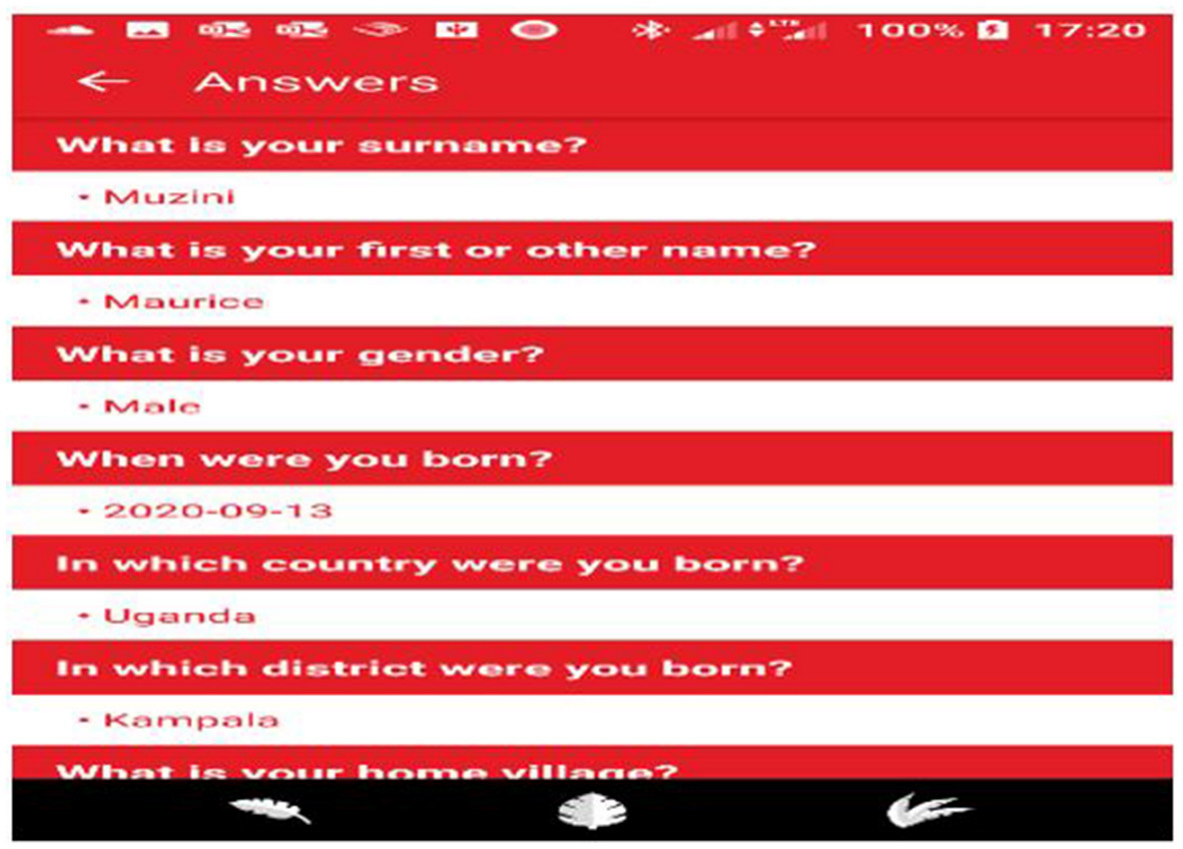
The SaniDigS data collection platform.

**Figure 3:**
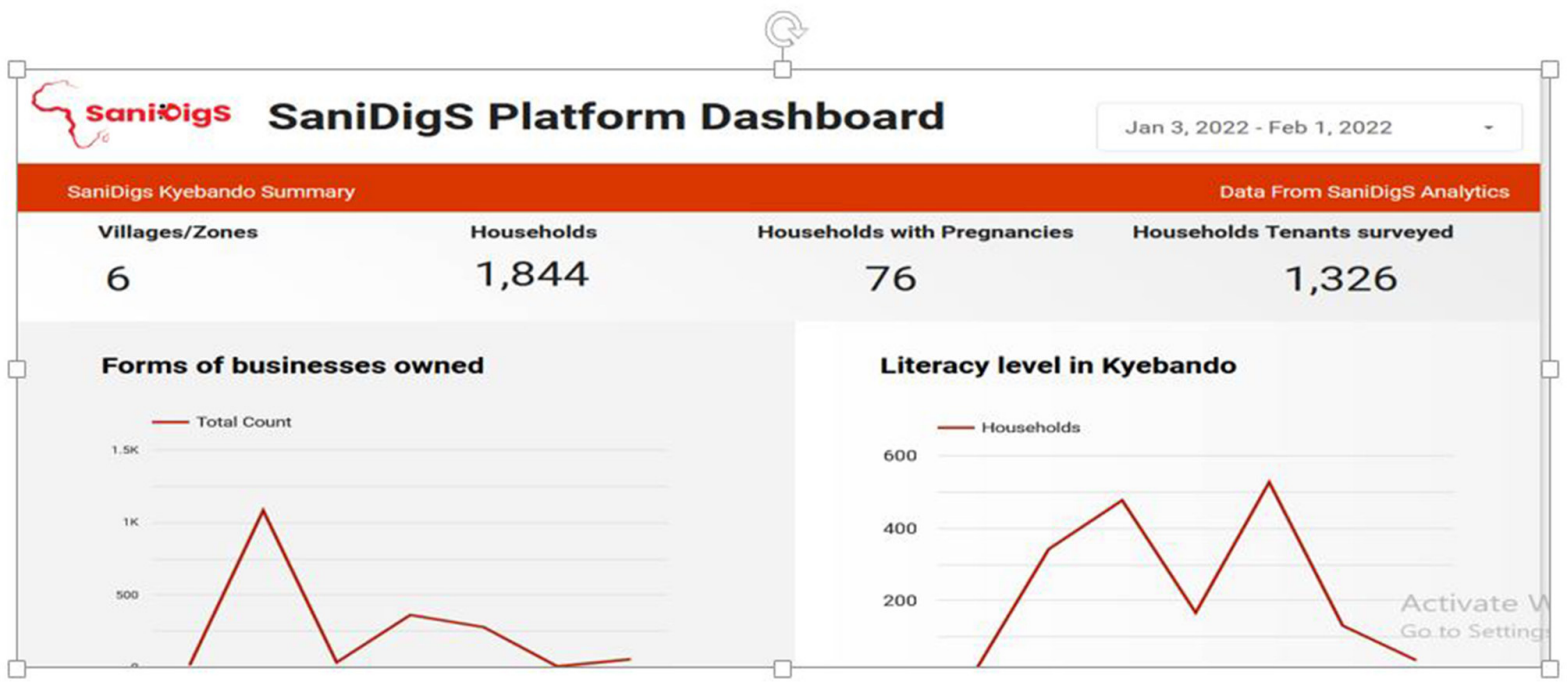
SaniDigS platform dashboard.

**Figure 4:**
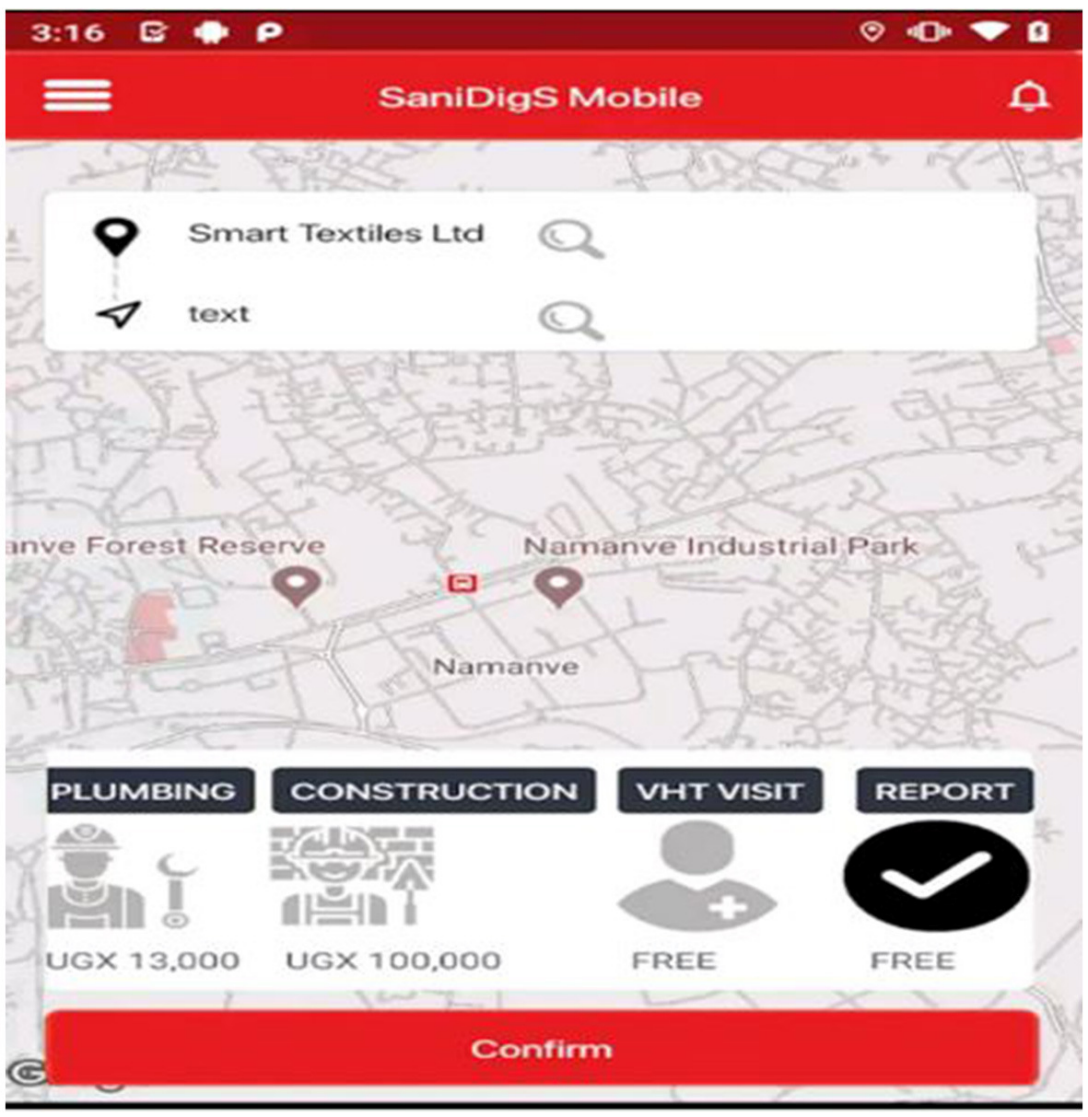
The SaniDigS mobile app indicating the services and cost.

**Figure 5:**
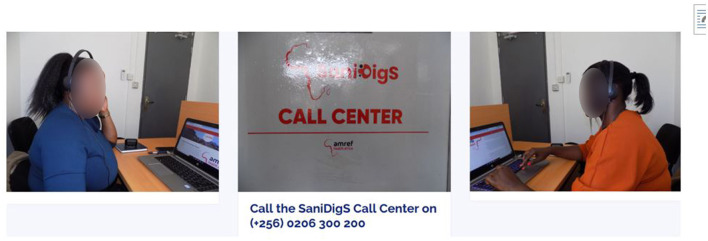
Attendants of the SaniDigS call center.

**(a) The SaniDigS Champion Application:** This app was designed for data collection. This was birthed from the need for high quality data for SaniDigS project to perform analytics. In as much as SaniDigS was initially designed to capture sanitation data, the stakeholder analysis meetings expressed their interest in a system that would capture holistic data beyond just sanitation. This field App uses the wizard fashion (question by question) of data collection, allows the data collector to go back to the question and make change in case of any errors, a capability provided by the back button (arrow at the top) and forward with the “Next” button. Some of the variables captured in this app include age of respondents, sex, date of birth, and sanitation status among others (Figure 2).

(**b) The SaniDigS platform dashboard:** This platform allows quantitative data visualization. It provides quick analytics and visualizations in terms of bar charts, line graphs, and numbers. This provides policy makers and planners with real time data and trend analysis for informed decision making. As a pilot the household survey data was uploaded on the dashboard to enable demonstration of the visualization capabilities and enable public use of the date for planning and further inquiries (Figure 3).

(**c) The SaniDigS mobile application**. This connects the service providers and the community. The community is able to access sanitation services and products from qualified service providers. The services include garbage collection, fumigation, plumbing, latrine construction and health worker (VHT) visit. The app also displays the different service providers, their services and location (Figure 4).

(**d) Call center:** The call center handles any inquiries, manages user expection, and also links the community to service and product providers (Figure 5).

## 4 Discussion

Through a user centered design, we aimed to document SaniDigS implementation, user needs and design features of the SaniDigS-a sanitation digital solution that aimed to provide real time access to sanitation data and services for better planning, coordination and access to sanitation services in Kawempe division, Kampala Uganda. From the phase of “understanding the context” the household survey showed that majority of the households were using an ordinary pit latrine with a quarter of them being full and 85.5% of the households lacked hand washing facilities. Such household characteristics are typical of urban poor communities that would need access to a sanitation solution that is easy to use, low cost, and able to connect clients to service providers and market the products that can facilitate improvement in sanitation status (as identified in phase 2 on “user needs”). The SaniDigS innovation was co designed to include four features including SaniDigS Champion App, dashboard, mobile App and the call center as key features to meet the characteristics of the household members in Kawempe.

In this study it was indicated that a number of households lacked access to basic sanitation services with a number of them having filled up pit latrines. The several filled up latrines were attributed to the lack of access to emptying services while others noted construction and emptying of pit latrines being costly. The policy makers reported the lack of real time data for better planning and coordination which led to inability to plan for timely reconstruction of public latrines to ease pressure on the few community-owned latrines. Other studies in Sub Saharan Africa have similarly observed that in urban slum areas, most households lack access to basic sanitation services, due to poverty, but also lack of access to service providers (4, 24, 25). Such challenges expose communities to sanitation related diseases and outbreaks which underscores the need for linking households to service providers with subsidized sanitation services like latrine construction products. It is also important to have real time data for better decision making and planning for better access to sanitation services in urban slums (26).

Making connections between the needs of users and the potential innovation to be able to meet those needs is critical for a successful design and implementation of an innovation (27). It is emphasized that there is need for more interactive engagement between users and producers (27) and in this study we engaged key stakeholders including the community, policy makers and service providers to identify user needs for SaniDigS. The community members emphasized the need for the innovation to be easy to use without internet, low cost of sanitation products and of good quality. These have been highlighted in literature to be critical success factors for community innovations (28).

The need for a non-internet dependent innovation is critical given the fact that only few Ugandans and other urban dwellers in African countries have access to internet (29). Lack of internet connectivity has been reported to frustrate innovations especially in remote areas (29). Therefore, there is need to think of SaniDigS apps or design features or elements which can be easily accessed offline for example the locations of the sanitation service providers and their contacts

The cost of products and services is a key determinant of consumption of services and services. In this study low cost was one of cited user needs for SaniDigS. This underscores the need to engage service and product providers in the co-design of the solution to ensure that services and products through SaniDigS are subsidized to encourage community uptake. The service and product providers also need to ensure that the services and products satisfy the customers' expectations through timely response and genuine sanitation products as identified in the user needs assessment in Table 3. In response to ensuring quality services, SaniDigS designed the mobile application to ensure that clients with smart phones can easily access the providers and sanitation products and through the call centers, internet and non-internet users could also access products and services, and express their dissatisfactions with the services and products of the different providers.

The service providers expressed the need for SaniDigS to be a ble to advertise, market their products and create more connection to consumers. This makes business sense which could attract more providers to use the platform but also ensure sustainability. Therefore, the design included a mobile app with a popup feature where providers could advertise their products/services and include a price list. This implies that everyone with a smart phone is able connect with any service providers. With the increasing use of smart phones in the country, this provides an opportunity for SaniDigS to succeed and have a wider reach.

The policy makers noted the need for the sanitation innovation to ensure that it provides real time, comprehensive data and be able to visualize to provide quick evidence for decision making. Through co designing of SaniDigS with policy makers, the SaniDigS platform dashboard was in-built to facilitate visualization of sanitation data in simple formats such as graphs. The dashboard acts as one stop center where data can easily be accessed for planning and decision making. The SaniDigS champion app was also adjusted to comprehensively capture the data beyond sanitation to include variable like common illnesses, HIV status, as per needs of the policy makers. Sanitation promotion is enhanced by the interface for different stakeholders along the sanitation chain on one centralized dashboard. In this way, SaniDigS has the potential to improve multi-sectorial decision making, planning, and provision of safely managed sanitation services to -urban settlements.

Through the engagement of policy makers, the design process of SaniDigS was informed by existing sanitation laws and policies such as the Public Health Act of Uganda that forbids the omission of nuisances of any kind which include but are not limited to indiscriminate disposal of solid waste, fecal matter and wastewater and demands that everyone must have a safe pace for the disposal of fecal matter and must. The other laws that were considered are the draining and sanitation rules that stipulate that every household and/or premises must have a sanitary facility and the grade two building rules that demand for provision of standard sanitation services by the owners of premises. Consideration of the policy environment is important to ensure scale up and sustainability of innovations.

### 4.1 Study limitations and strengths

The study was limited by the fact that the sanitation digital innovation concept was new to the users and there is possibility that we did not capture the breadth and depth of user needs since we consulted only a sample of respondents and stakeholders. However, this was minimized by the iterative nature of the design processes which included a number of workshops and multiple data sources for triangulation. It was difficult to adopt a few of the user needs such as an offline app. However, the solution design was adapted to cater for non-internet users by including a call center where users could call in any time to access the services without requiring internet. The dashboard was also designed to capture real-time data which indicated positive trends in the matrixes of interest including turnaround time in service and product delivery, growth of business in numbers and size, the use of the service by the community health workers who used the platform to pick information for decision making. Particularly, this was seen when the community health workers used the platform to pick which communities were mostly calling for fecal sludge emptying services. Also, fecal sludge emptying was being done but the sludge was not reaching the treatment plant as indicated by discrepancies in volumes documented at the plant which prompted the community health workers to intervene.

During the piloting stage, the SaniDigS team, took the opportunity during the household survey phase not only to collect sanitation data to improve the platform but also to promote the benefits of what SaniDigS will be bringing to the members and the community at large. We observed our target market had a division of languages for communication (English and Luganda), we then proceed to dedicate call agents for the two languages respectively. The client base was also skeptical of the quality of the WASH products such as briquettes and we had work out a way for the households to receive samples and understand the benefits of using renewable cooking fuel briquettes over charcoal. Lastly, on the side of service providers especially the ones providing the toilet emptying service, we had to remind them every morning to turn on their availability signal in the SaniDigS app such that they can accept emptying requests. This arose as client called through the call center and complained that the emptiers were not responding to their requests.

## 5 Conclusion

This paper describes a sanitation digital solution (SaniDigS) design and implementation using an UCD approach. Through the description of the context (sanitation situation of the community), identifying user needs and co-design of the solution with users, we were able to identify the design features of SaniDigS that would best meet the various users of the solution and how to combine the design features into a system or platform.

Through SaniDigS, there is hope that we could solve the challenge of lack of real time data for planning, decision making and access to sanitation services and products. SaniDigS was co designed factoring in a number of user needs which increases the chances for usability and sustainability. Further research needs to be conducted to evaluate the effects of SaniDigS on planning, coordination and access to sanitation services and products and to inform other modifications like the offline app features.

## Data availability statement

The raw data supporting the conclusions of this article will be made available by the authors, without undue reservation.

## Ethics statement

This study was approved by Mildmay Uganda research and Ethics Committee (MUREC) and Ethics Committee (protocol number REC REF 0112-2020) and the Uganda National Council of Science and Technology (UNCST). This stakeholder consultation process and interviews adhered to the minimum ethical standards of (1) anonymous data collection strategies to maintain a high level of confidentiality; (2) the household interviews were asked to participate voluntarily; (3) written informed consent was sought; and (4) no study materials contained names or other explicit identifiers of participants.

## Author contributions

CM and MW conceptualized the study. MN, PK, and MW participated in data collection. CM and MN drafted the first manuscript. CM, MN, MW, and PK reviewed the first manuscript draft. All authors read and approved the final manuscript.
